# Immune cell infiltration landscape and immune marker molecular typing in preeclampsia

**DOI:** 10.1080/21655979.2021.1875707

**Published:** 2021-02-04

**Authors:** YiLin Meng, Chuang Li, Cai-Xia Liu

**Affiliations:** a Department of Gynecology and Obstetrics, Shengjing Hospital of China Medical University, Shenyang, Liaoning Province, China; b Key Laboratory of Maternal-Fetal Medicine of Liaoning Province, Shenyang, Liaoning Province, China

**Keywords:** Preeclampsia, immune infiltration, immune-related gene, differential analysis, WGCNA, network mining, protein interaction network, disease typing

## Abstract

Preeclampsia (PE) is an important topic in obstetrics. In this study, we used weighted gene co-expression network analysis (WGCNA) to screen the key modules related to immune cell infiltration and to identify the hub genes for the molecular subtyping of PE. We first downloaded a set of PE transcriptional data (GSE75010; 157 samples: 80 PE and 77 non-PE) from the GEO database. We then analyzed the PE samples and non-PE samples for immune cell infiltration and screened cells with differences in such infiltration. Next, we downloaded the immune-related genes from an immune-related database to screen the expression profile of the immune-related genes. Then, we obtained a candidate gene set by screening the immune-related genes differentially expressed between the two groups. We used WGCNA to construct a weighted co-expression network for these candidate genes, mined co-expression modules, and then calculated the correlation between each module and immune cells with differential infiltration. We screened the modules related to infiltrating immune cells, identified the key modules’ hub genes, and determined the key module genes that interacted with each other. Finally, we obtained the hub genes related to the infiltrating immune cells. We classified the preeclampsia patients by unsupervised cluster molecular typing, determined the difference of immune cell infiltration among the different PE subtypes, and calculated the expression of hub genes in these different subtypes. In conclusion, we found 41 hub genes that may be closely related to the molecular typing of PE.

## Graphical abstract

**Figure:**
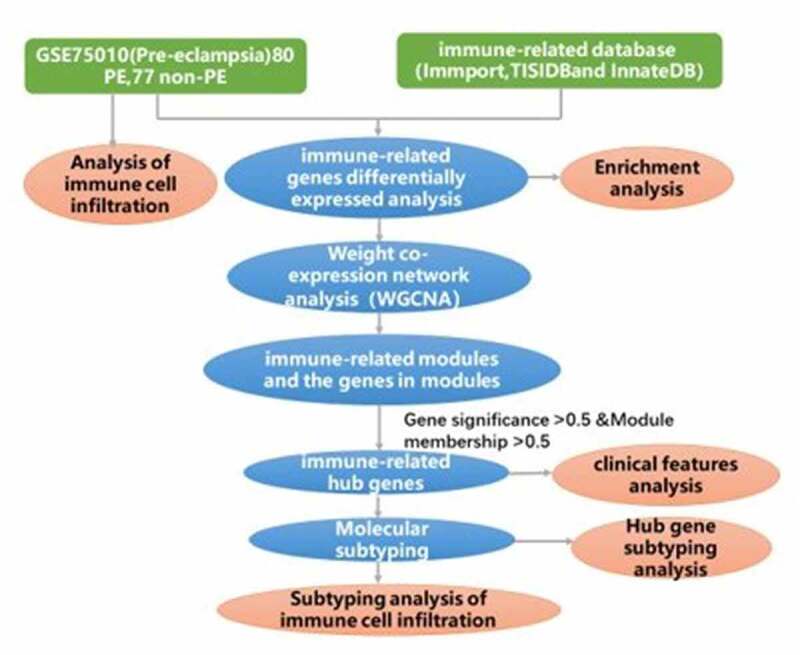

## Introduction

Preeclampsia (PE) is a pregnancy-specific hypertensive disease, in which individuals with normal blood pressure before pregnancy develop hypertension, urinary protein, and other features after 20 weeks of pregnancy [1]. PE affects 3% to 5% of women worldwide and is a major cause of maternal death [2]; it can lead to iatrogenic preterm birth and fetal growth restriction. PE also causes about 15% of preterm births worldwide [2]. The basic pathophysiological changes in PE are endothelial damage, systemic arteriospasm, and then a decrease of systemic perfusion and multiple organ damage, which seriously threaten the health of the mother and baby [3,4]. However, at present, its etiology and pathogenesis have not been fully elucidated, so this remains an important research topic in obstetrics. Many studies have suggested that the mechanism behind PE may be related to the interaction of multiple genetic factors and environmental factors. For instance, PE could occur in pregnant women suffering from basic diseases, such as hypertension, or having other high-risk factors, such as multiparity and older age [5,6].

Although the exact cause of PE remains unclear, its clinical manifestations are thought to be the result of endometrial dysfunction caused by placental dysfunction, characterized by inadequate uterine spiral artery transformation. This placental defect is most likely associated with partial breakdown of the mother’s immune tolerance [7]. At the maternal–fetal interface, which consists of decidual stromal cells, decidual immune cells, and trophoblast cells [8], the villi from the fetal trophoblasts are in close contact with the maternal vascular system, so healthy mothers need to develop immune tolerance to avoid immune attacks on fetal tissue and retain the ability to locally defend against pathogens [9,10]. At the same time, the extra trophoblast cells invade the decidua, reach the myometrium, participate in remodeling of the maternal spiral arteries, and convert some of the maternal uterine spiral artery endothelial cells into extra trophoblast cells [8].

Many studies using bioinformatics for screening to identify PE-related genes have been performed. Many important genes and pathways related to PE, including TLRs [11], the MAPK family [11], and inflammation and complement-related pathways [12,13], have been identified by determining the methylation levels of placental genes [11] and genes encoding serum proteins [13], urinary proteins [12], or PE family members [14] in patients with PE. However, the above research focused on the screening of biomarkers for diagnosing PE. Recently, immune cell infiltration has been used as an important feature to study the tumor immune microenvironment, to screen genes diagnostic for various tumors, and to help predict the appropriate treatment and prognosis of patients [15]. Meanwhile, in other non-tumor inflammatory diseases such as ulcerative colitis [16], applications for measuring immune cell infiltration have also been developed. In view of the importance of immune cell infiltration in the etiology of PE, a method considering immune cell infiltration should be useful for screening hub genes, which could play an important role in finding molecular markers of PE subtypes and for further optimizing therapy for this condition.

The aim of this study was to screen the key modules related to PE immune infiltration based on WGCNA and identify the hub genes for PE molecular subtyping. The differential expression of hub genes among different PE subtypes suggests that these genes are potential markers for PE subtyping.

## Materials and methods

### Downloading and preprocessing of data

First, we downloaded a set of PE chip data, GSE75010, from the GEO database (https://www.ncbi.nlm.nih.gov/gds), as shown in Table 1. We preprocessed the data as follows: The downloaded dataset was log2-transformed quantile-normalized signal intensity, the probes were first mapped to the gene, the null probes were removed, and multiple probes were mapped to the same gene.

**Table 1.** GEO database data of preeclampsia mRNA expression profile

Dataset ID	Platform	Preeclampsia	Normal
GSE75010	GPL6244	80	77

From Immport [17] (https://www.immport.org/), TITIIDB [18] (http://cis.hku.hk/TISIDB/), and INNATEDB [19] (http://www.innatedb.com), we obtained 2496 immune-related genes, which we then cross-linked with GSE75010 microarray data. Finally, we obtained the expression profiles of 922 immune-related genes.

## Analysis of immune cell infiltration

We obtained the R language source code and its given feature immune gene expression set from CIBERSORT [20] (https://cibersort.stanford.edu/index.php), namely, LM22.txt. We determined the immune cell infiltration of GSE75010 preeclampsia samples and normal samples and categorized different subtypes of preeclampsia as classified by hub genes.

## Screening and enrichment of differentially expressed genes (DEGs)

We used the limma package in R to analyze the differences of immune-related gene expression profiles between PE and non-PE in the downloaded microarray data. Upon applying a Benjamini–Hochberg false discovery rate (FDR)-corrected P value [21] of <0.05 as the threshold, we obtained 428 differentially expressed immune-related genes, of which 181 were upregulated in PE and 247 were downregulated. We then determined the associations of these genes with functions and pathways using Gene Ontology (GO) and Kyoto Encyclopedia of Genes and Genomes (KEGG) using the R package clusterProfiler.

## Weight co-expression network analysis

We used the R package WGCNA software to construct a weighted co-expression network of candidate differentially expressed immune-related genes. We found that the log(k) of the node with degree K had a negative correlation with the log[P(k)] of the probability of occurrence of the node, and the correlation coefficient was greater than 0.8, which was consistent with the scale-free network of the co-expression network. To ensure that the network was scale-free, we selected the optimal soft threshold of 5. In the next step, we transformed the expression matrix into an adjacency matrix, which we then converted into a topological matrix. Based on topological overlap matrix (TOM) [22], we clustered the genes using average linkage and Pearson’s correlation. We constructed a hierarchical clustering tree using dynamic hybrid cutting, setting the minimum number of genes per gene network module to 30. After determining gene modules by the dynamic shear method, we calculated the eigengenes of each module at one time, then clustered the modules, merged the nearby modules into new modules, set height equal to 0.25, and obtained five modules in total.

Then, we calculated Pearson’s correlation between the five modules and the clinical traits of differentially infiltrated immune cells. We found that the blue and brown modules were most associated with immune cell infiltration. We then calculated Pearson’s correlation coefficient in the most relevant blue and brown modules between immune genes and the modules and clinical features, respectively.

## Screening of hub genes

First, we selected the genes with a gene significance coefficient greater than 0.5 and a module membership coefficient greater than 0.5 as candidate hub genes. Then, we constructed a PPI network of the blue and brown modules and selected a connectivity threshold of 0.1, which led to the recognition of a total of 120 nodes. Finally, we obtained 41 critical hub genes that overlapped between the 49 hub genes and 120 nodes.

## Hub gene-mediated molecular typing of preeclampsia

We also performed disease classification of the PE samples by using the identified hub genes and determined the optimal K value (number of classes) by searching for the best SSE. We classified PE into different subclasses by unsupervised clustering K-means combined with TSEN dimension reduction. We examined the expression patterns of these hub genes in the different subclasses and analyzed the significantly differentially expressed genes in the different subclasses (Kruskal–Wallis, P < 0.05). These differentially expressed genes are potential marker genes of PE subclasses.

## Statistics and data visualization

All of the analyses were performed using R (V. 4.0.2) statistical software. The differentially expressed genes were calculated using the R package limma. The enriched functions associated with the differentially expressed genes were determined using the R package clusterProfiler [23]. The co-expression network was constructed using the R package WGCNA. Computation of Spearman’s correlation and the bi-directional detection were carried out using the function cor.test. t-Distributed stochastic neighbor embedding (T-sne) was performed through the R package Rtsne.

## Results

In this study, we analyzed microarray datasets from the GEO database. We used Cell-type Identification by Estimating Relative Subsets of RNA Transcripts (CIBERSORT) to analyze differentially infiltrating immune cells in PE. We also analyzed the biological processes and signal transduction pathways related to the DEGs, and evaluated the ability of gene signatures to predict the PE subclass. Here, we screened 41 critical hub genes by comparing the immune cell infiltration and classified the samples into five subclasses according to the hub genes. We found that most of the genes were expressed at a low level in cluster 1 and a high level in cluster 2, suggesting that these genes are potential markers in different PE subtypes.

## Differences in immune cell infiltration between preeclampsia and normal subjects

**Figure 1.**
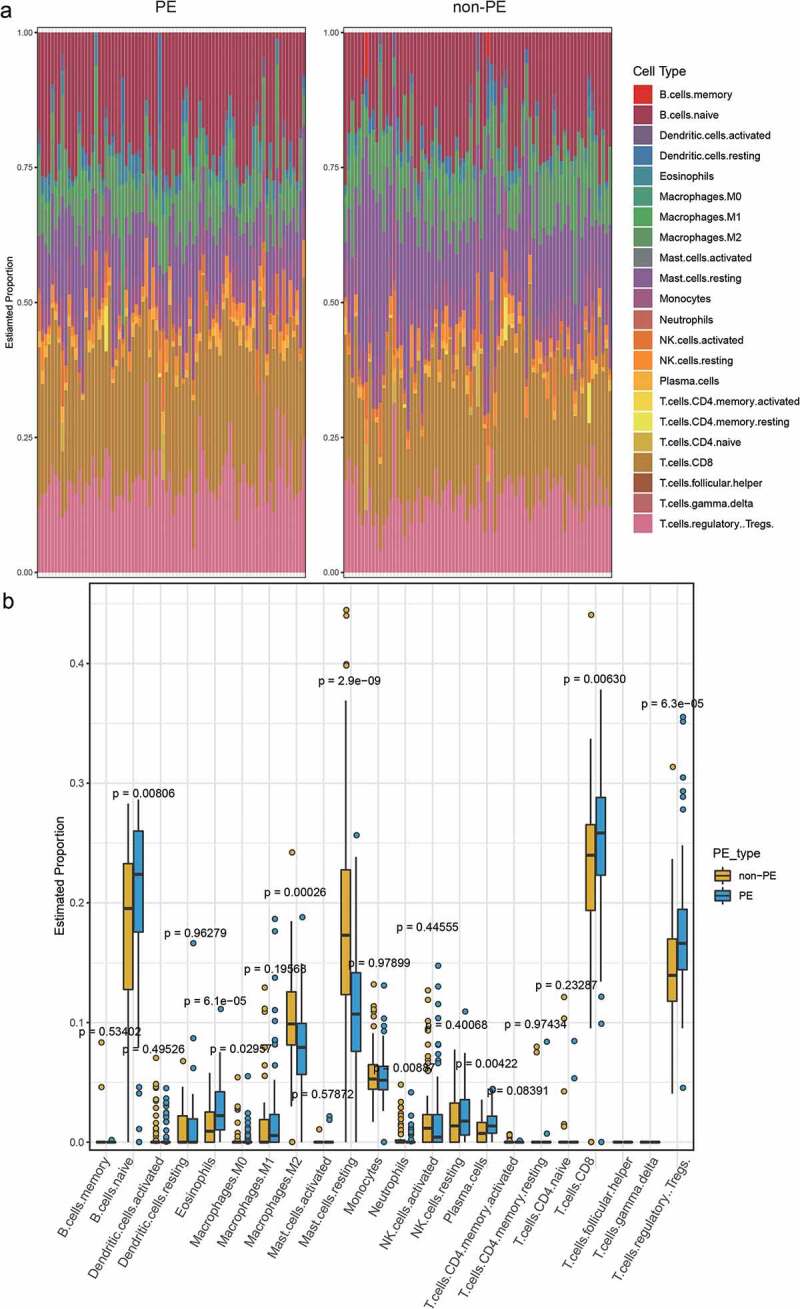
A. The immune cell infiltration in PE and normal samples. B. The difference of immune cell infiltration between PE and normal samples (Wilcoxon’s test, p < 0.05)

Using the CIBERSORT LM22.txt file as the feature gene set, we analyzed the immune cell infiltration in PE and normal samples. The results showed that the infiltration of PE samples was significantly different from that of normal samples (p < 0.05) regarding B cells naive, plasma cells, T cells CD8, T cells regulatory (Tregs), Macrophages M0, Macrophages M2, mast cells resting, eosinophils, and neutrophils (Figure 1).

## Screening and functional enrichment of differentially expressed immune-related genes

**Figure 2.**
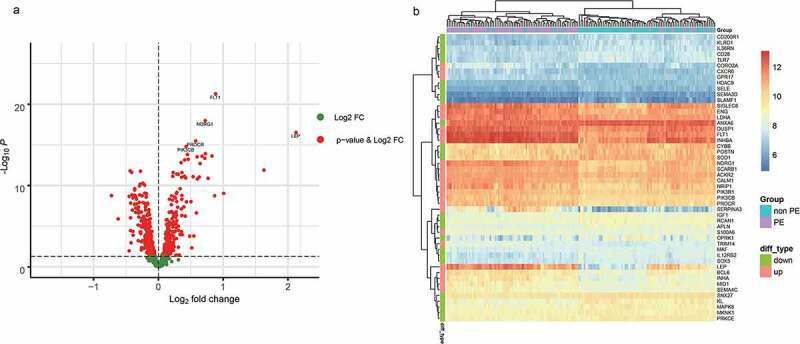
A. The differentially expressed immune-related genes (volcano plot). B. The 25 most differentially expressed upregulated and downregulated immune-related genes were selected to compare their expression differences

Setting p < 0.05 as a threshold, we obtained 428 differentially expressed immune-related genes, of which 181 were upregulated in PE and 247 were downregulated (Figure 2; Supplementary Table 1).

**Figure 3.**
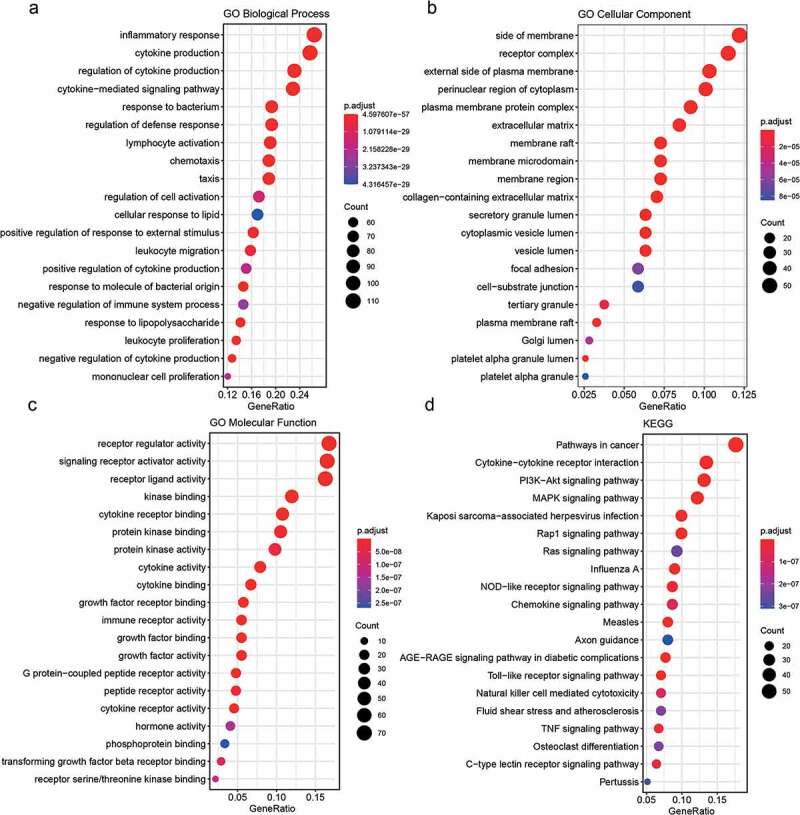
Functional enrichment results of differentially expressed immune-related genes

For the 428 differentially expressed immune-related genes, we performed GO and KEGG enrichment analyses, and found that these genes were particularly associated with immune-related functions, such as inflammatory response, cytokine production, cytokine–cytokine receptor interaction, and biological functions related to the MAPK immune signaling pathway (Figure 3).

## Construction of co-expression networks based on differentially expressed immune-related genes

**Figure 4.**
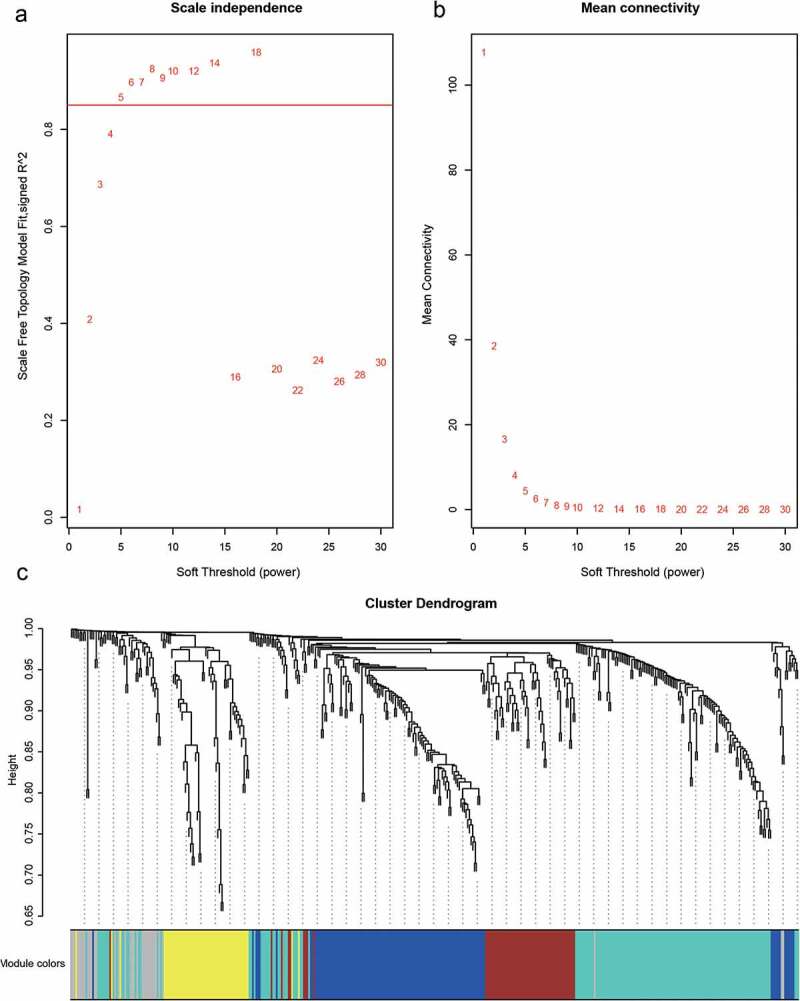
A, B. Analysis of network topology for various soft-thresholding powers. C. Gene dendrogram and module colors

We used the R package WGCNA software to construct a weighted co-expression network of candidate differentially expressed immune-related gene sets. We found that the log(k) of the node with degree K had a negative correlation with the log[p (k)] of the probability of occurrence of the node, and the correlation coefficient was greater than 0.85, which is consistent with the scale-free network of the co-expression network. To ensure that the network was scale-free, we selected the optimal soft threshold of 5 (Figure 4a,B).

The next step was to transform the expression matrix into an adjacency matrix, which we then converted into a topological matrix. Based on TOM, we used the average-linkage hierarchical clustering method to cluster genes according to the criteria of the hybrid dynamic shear tree and set each gene network module to at least 30 genes. After determining gene modules by the dynamic shear method, we calculated the eigengenes of each module at one time, and then clustered the modules, merged the nearby modules into new modules, and set height equal to 0.25. This led to five modules being obtained (Figure 4c). We counted the number of genes in each module, as shown in Table 2, where 428 genes were distributed among the five modules (Table 2).

**Table 2.** Gene statistics corresponding to each module

Module	No. of Genes
blue	120
brown	62
gray	34
turquoise	157
yellow	55

## Screening key modules related to differentially infiltrating immune cells

**Figure 5.**
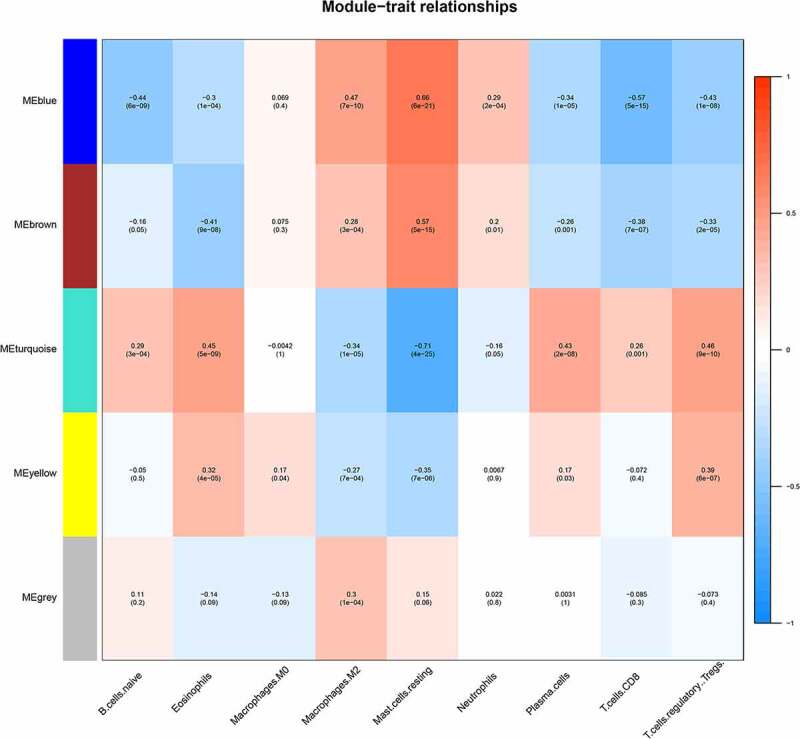
Module–trait relationships

We selected the differentially infiltrating immune cells shown in Figure 1 as sample traits and calculated Pearson’s correlation coefficient between module eigengenes (ME) and sample traits for each module (the higher the representative module’s Pearson’s correlation coefficient, the more important it is). Each row of Figure 5 shows the feature vector genes of each module, and each column shows the sample information of different immune cells. From red to blue, the correlation between eigengenes and sample characteristics decreases. The numbers in each box represent the correlation coefficient between the gene module and the corresponding feature, and the values in parentheses represent the p values. In this figure, it can be seen that the blue and brown modules are most closely related to the phenotype of differentially infiltrating immune cells.

## Identifying key module hub genes

**Figure 6.**
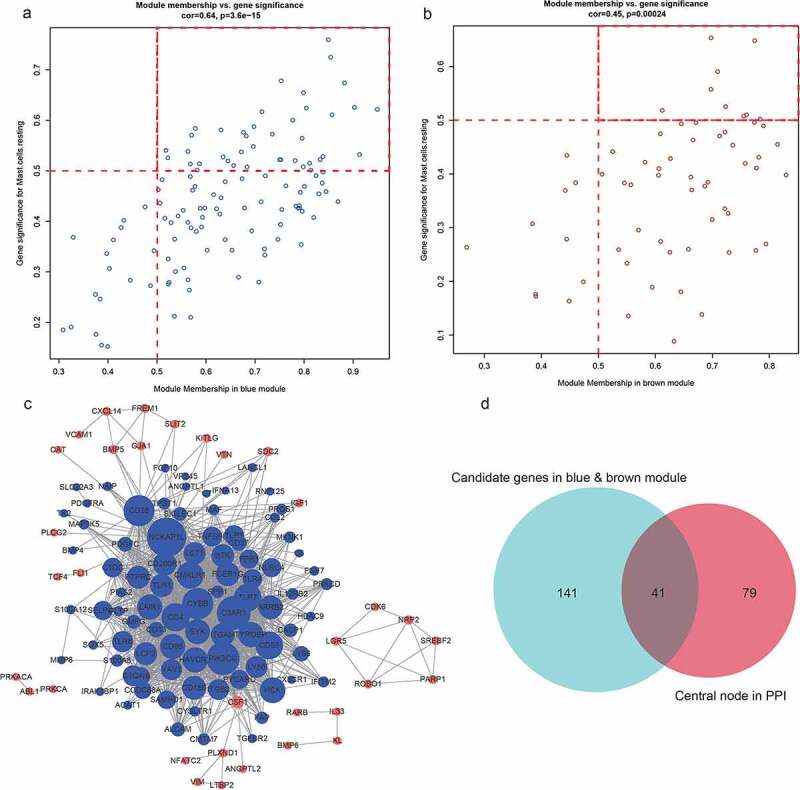
A, B. The genes and modules of the blue and brown modules and the correlation of different immune cell characteristics. C. The interaction network of the blue and brown modules. In the figure, blue represents the genes of the blue module, brown represents the genes of the brown module, node size represents the degree of the node. D. Venn diagram of the candidate hub gene and PPI network graph

We identified a total of 182 immune-related genes from the most relevant blue and brown modules. Next, we calculated the correlation between these 182 immune-related genes and the modules and their phenotypes (resting mast cells). We selected the genes with a gene significance coefficient greater than 0.5 and a module membership coefficient greater than 0.5 as the key genes to identify 49 potential hub genes, of which 40 were blue and 9 were brown (Figure 6a, B). We then constructed a PPI interaction network (Cytoscape 3.6.0 http://www.cytoscape.org/) based on blue and brown modules (Supplementary Table 2) with a connectivity threshold of 0.1 to identify 120 node nodes (Figure 6c, Supplementary Tables 3 and 4). The 49 candidate hub genes were then surveyed to determine those overlapping with 120 module genes to obtain 41 final hub genes. In this way, we cross-referenced 41 hub immune-related genes, including 33 in blue and 8 in brown (Figure 6d, Supplementary Table 5).

## Analysis of correlation between hub genes and clinical features

**Figure 7.**
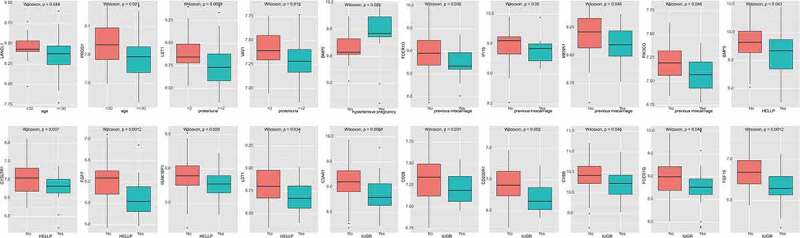
Correlation between hub genes and clinical features

From the clinical features downloaded from GEO, we analyzed the correlation of these 41 hub genes with the clinical features of advanced-age pregnancy, maternal hyperproteinuria, maternal hypertension, maternal abortion, HELLP syndrome, and intrauterine growth retardation (IUGR). The results showed that LANCL1 and PROS1 may be associated with advanced-age pregnancy; LST1 and VAV1 may be associated with maternal hyperproteinuria; BMP5 may be associated with maternal history of hypertension; FCER1G, IFI16, MKNK1, and Pik3cg may be associated with maternal history of abortion; bMP5, Cysltr1, FGF7, IRAK1BP1, and LST1 may be associated with HELLP syndrome (hemolysis, elevated liver enzymes, and low thrombocytopenia); and c3AR1, CD200R1, CD28, CYBB, FCER1G, FGF10, IFI16, IL12RB2, Kitlg, LGR5, NCKAP1L, PIK3CG, Rarb, TLR7, and VAV1 may be associated with IUGR (Figure 7; Supplement Figure 1).

## Unsupervised cluster molecular typing of disease samples based on hub genes

**Figure 8.**
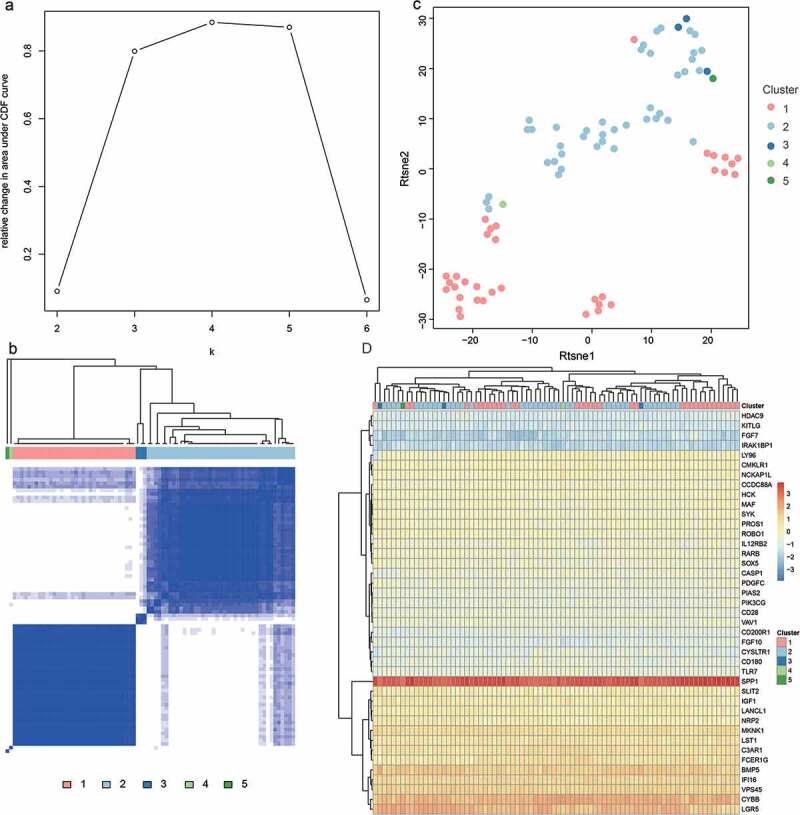
A. Use of SSSE to find the best inflexion. B. The cluster of PE subtypes. C. Based on Tsne to show PE subtypes. D. The expression of 41 hub genes in PE subtypes [log2(EXP + 1) scale]

Based on the analysis results of the WGCNA co-expression modules, we further analyzed 41 hub genes identified by blue and brown modules and subjected them to unsupervised clustering. We selected PE samples (N = 80) to extract the expression profile data of these 41 hub genes. We classified all PE samples by the K-means unsupervised clustering method. First, we selected the optimal K value by searching for the inflexion of the sum of squares due to error (SSE, means the sum of squares of the distances from all points to the center of the cluster). As shown in Figure 8a, the curve declines slowly between K = 4 and 5, so we chose K = 5. Figure 8b shows a consistent cluster of different subtypes of PE samples. We used Rtsne to reduce the amount of gene expression data, as shown in Figure 8c; all PE samples can be clearly divided into five categories, combined with co-expression of key genes in all PE (Figure 8d), although there were no taxonomic differences based solely on the expression of individual genes, the combination of all co-expressed key genes also showed a high degree of agreement with Figure 8c. Therefore, we can infer that the 41 co-expressed genes are of great significance to the typing of PE.

## Difference of immune cell infiltration in different PE subtypes

**Figure 9.**
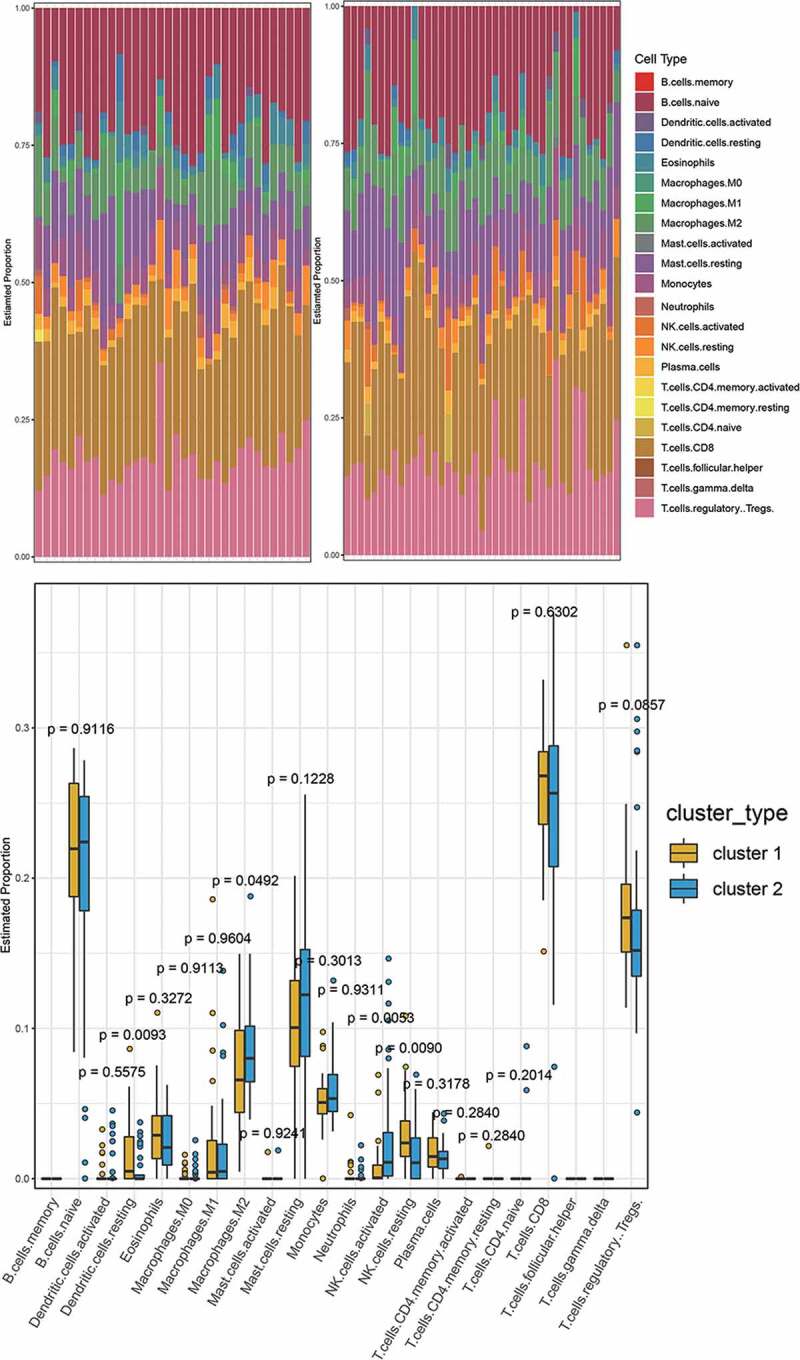
A. The immune cell infiltration in cluster 1 and cluster 2. B. The difference of immune cell infiltration between cluster 1 and cluster 2 (Wilcoxon’s test, p < 0.05)

Using LM22.txt obtained from the official CIBERSORT website, we analyzed cluster 1 and cluster 2 for immune cell infiltration. The results in Figure 9 show that there were significant differences between the cluster 1 and cluster 2 subtypes (p < 0.05) in four types of immune cell, namely, dendritic cells resting, macrophages M2, NK cells resting, and NK cells activated, revealing that the 41 hub genes that we identified could help in the typing of preeclampsia patients.

## The difference of hub gene expression in the different PE subtypes

**Figure 10.**
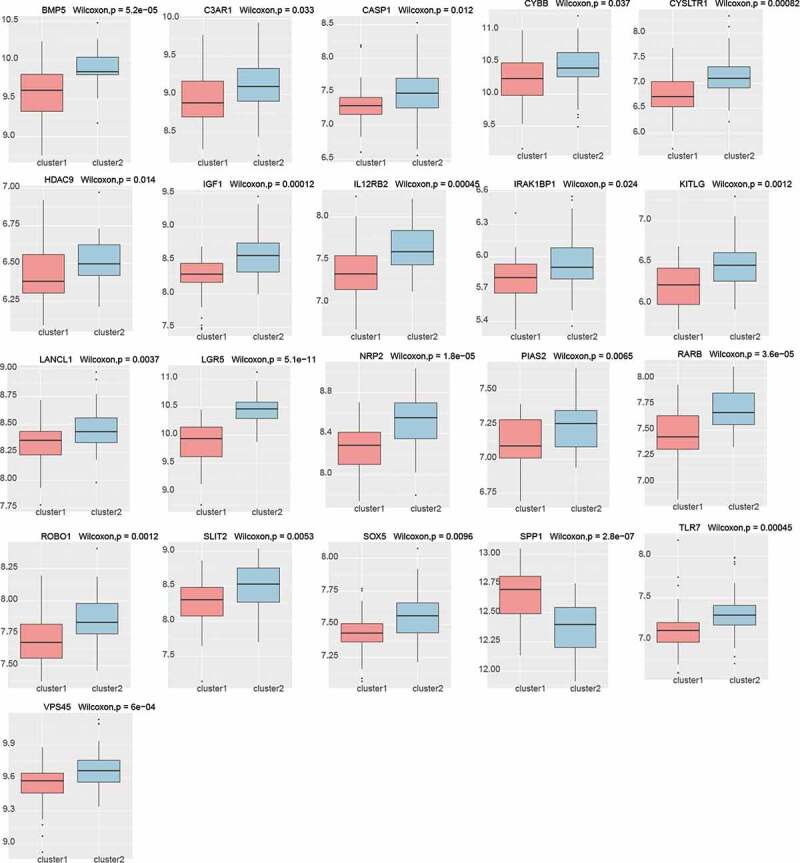
Differential expression of hub genes in different PE subclasses (21/41, Kruskal–Wallis, p < 0.05)

To explore the expression of these genes in the different PE subclasses, we calculated the expression of the hub genes in the different preeclampsia subtypes based on Figure 9, and found that most of the genes (21/41) were expressed differentially (Figure 10, Kruskal–Wallis, p < 0.05). Except for SPP1, most genes were expressed at a low level in cluster 1 and at a high level in cluster 2. It is suggested that these genes may serve as marker genes in different types of preeclampsia.

## Discussion

As a hypertensive disorder complicating pregnancy, PE is one of the leading causes of maternal mortality globally. PE-induced fetal growth restriction, preterm birth, and other common complications also place huge economic and psychological costs on the affected family and society at large. Maternal immune tolerance, which plays an important role in the pathogenesis of PE, and the remodeling of spiral arteries are closely related to the infiltration of placental immune cells. Immune cell infiltration is a novel bioinformatic approach that has been used to investigate the diagnosis and prognosis of a variety of diseases including colon [24], gastric [25], and breast cancer [26], as well as ulcerative colitis [16]. Although an increasing number of studies have actively sought molecular markers for the diagnosis and treatment of PE via data mining and analysis in GEO or other databases, study of the landscape of immune cell infiltration in PE and immunomarker molecular typing are yet to be performed.

Here, we obtained the gene expression profiles of 7,859 genes by filtering the gene chip data downloaded from GEO. We obtained 2496 immune-related genes from the immune-related database and 922 genes with expression profile data. We screened a total of 428 differentially expressed immune-related genes (181 upregulated in PE, 247 downregulated, p.adj < 0.05) using LIMMA. We used the R package clusterProfiler for enrichment analysis of these differentially expressed genes and found that they were linked to immune-related functions such as inflammatory response, cytokine production, cytokine–cytokine receptor interaction, and immune signaling pathway-related biological functions (MAPK signaling pathway). This is consistent with previous studies that reported that the above biological processes and pathways play important roles in the development of PE. Using proteomic techniques, it has been shown that proteins related to lipid metabolism and inflammatory pathways can be used as early diagnostic markers of PE [13]. In addition, in the literature, it was reported that, upon combining gene expression data of placental cells cultured in vitro with the GEO database, three hub genes diagnostic of PE, including MAPK13, were identified [11]. Reports have also been published on the involvement of the MAPK family in PE [27]. In a study by Ding et al., a total of 294 PE differentially expressed proteins (DEPs) were identified using urinary protein profiles; of these, the most differentially expressed proteins were involved in the complement pathway [12]. In conclusion, the results of functional annotation and pathway enrichment analysis confirmed that the immune process plays a key role in the pathogenesis of PE.

Using WGCNA to construct a weighted co-expression network of these genes, four co-expression modules were obtained, among which the immune-related genes of the blue and brown modules were those most strongly related to the infiltrating immune cells; the immune-related genes of the blue module numbered 120, while those of the brown module numbered 62. Further analysis of the genes of the most relevant blue and brown modules and the genes of the PPI interaction network that was constructed yielded 41 hub genes. These hub genes included LANCL1 and PROS1, which may be associated with advanced-age pregnancy; LST1 and VAV1, which may be associated with maternal hyperproteinuria; BMP5, which may be associated with a maternal history of hypertension; FCER1G, IFI16, MKNK1, and Pik3cg, which may be associated with a maternal history of abortion; BMP5, CYSLTR1, FGF7, IRAK1BP1, and LST1, which may be associated with HELLP syndrome (hemolysis, elevated liver enzymes, and low thrombocytopenia); and C3AR1, CD200R1, CD28, CYBB, FCER1G, FGF10, IFI16, IL12RB2, KITLG, LGR5, NCKAP1L, PIK3CG, RARB, TLR7, and VAV1, which may be related to intrauterine growth restriction (IUGR).

Among these, BMP5 [28], CD200R1 [29], CD28 [30], and TLR7 [31] have been shown to be significantly differentially expressed in PE. This provides further confirmation of the robustness of the current study. Meanwhile, the TLR family and associated pathways, including TLR7, have been shown to play important roles not only in PE but also in IUGR [32,33]. This is consistent with the results obtained in this study. It has also been shown that the mice treated with the TLR7/8 agonist CLO97 exhibited pregnancy-dependent hypertenia, endothelial dysfunction, splenomegaly, and placental infection similar to the features of PE [31]. Moreover, TLR7 and TLR9 levels on the vascular wall in mice infected with virus increased and ultimately caused placental growth retardation and intravascular growth restriction [32]. Interferon-inducible protein 16 (IFI16) is an innate immune receptor for intracellular double-stranded DNA (dsDNA) [34]. Li et al. found that its expression was significantly increased in the placenta of PE patients, and that activation of the IFI16 gene promoted the production of preeclampsia-related antigenic factors sFlt-1 and sEng in trophoblast cells [35]. Furthermore, studies have shown that changes in IFI16 gene expression in bovine endometrium lead to decreased embryo survival, further leading to miscarriage [36]. The above literature suggests that IFI16 not only plays a role in PE but also has a close relationship with abortion, which is consistent with our results.

In addition, immune cell infiltration was analyzed in preeclampsia and normal samples by CIBERSORT. Nine immune cells, namely, B cells naive, plasma cells, T cells CD8, T cells regulatory cells (Tregs), macrophages M0, macrophages M2, mast cells resting, eosinophils, and neutrophils, were screened. We found significantly different infiltration between cluster 1 and cluster 2 of four types of immune cell: dendritic resting cells, macrophages M2, NK cells resting cells, and NK cells activated cells. The literature shows that decidual NK cells promote trophoblast invasion by secreting chemokines, and decidual macrophages act as antigen-presenting phagocytes, secreting cytokines, and regulating the immune balance between mother and fetus [37]. T cells and dendritic cells (DCs) have been considered to be the key cells in regulating the immune balance [8,38]. This further illustrates the importance of immune cell infiltration in the pathogenesis and typing of PE.

According to the expression of the 41 identified hub genes in patients with PE in this study, we classified the samples by the K-means unsupervised clustering method, leading to the samples being grouped into five categories. We found that most of the genes were expressed at a low level in cluster 1 and a high level in cluster 2, suggesting that these genes are potential markers in different subtypes of preeclampsia. This parallels a study by Liu et al., in which EGR1, LEP, and HBB were used as DEGs to identify early-onset and late-onset PE [39]. In addition, in a study by Leavey et al., PE was classified into five categories based on placental gene expression using unsupervised clustering; it is thus anticipated that PE can be divided into different subclasses in the search for biomarkers for early diagnosis and to develop etiological treatment for specific subclasses [40]. The establishment of five categories in this previous study matches the number of clusters in the present work. Although different classification methods were used, the fact that PE was also divided into five clusters here indicates the feasibility of further subclassifying PE and adopting different treatment methods for different subtypes of this condition.

This study also has some shortcomings. First, although PE was divided into five clusters according to the hub genes, the numbers of samples in clusters 3, 4, and 5 were too small to be used for infiltration analysis. In future work, the sample size should be expanded and a more complete database should be established, enabling further analysis of the other clusters. Second, although 41 hub genes were identified as potential biomarkers for PE immunotyping, no in vivo or in vitro studies were carried out, so this should be a focus in future work.

## Conclusion

Using WGCNA, we divided the genes specifically differentially expressed in PE into four modules. We selected the two modules with the highest correlation with immune cell infiltration for a PPI network and obtained 41 hub genes. We classified PE samples into five clusters according to the unsupervised clustering of hub genes and found that cluster 1 and cluster 2 had significant differences in immune cell infiltration and hub gene expression. This work suggested that hub genes can be used to classify PE into different subtypes.

## Supplementary Material

Supplemental Material

## Article Highlights

We made the immune cell infiltration analysis of Pre-eclampsia (PE).

We screened 41 immune-related hub genes for PE.

The 41 hub genes are related to the molecular typing of PE.
