# Supplementary figures and images for: Immune cell infiltration landscape and immune marker molecular typing in preeclampsia
Source: Bioengineered. 2021 Feb 4;12(1):540–54. doi: 10.1080/21655979.2021.1875707 (PMC8806319; doi:10.1080/21655979.2021.1875707)

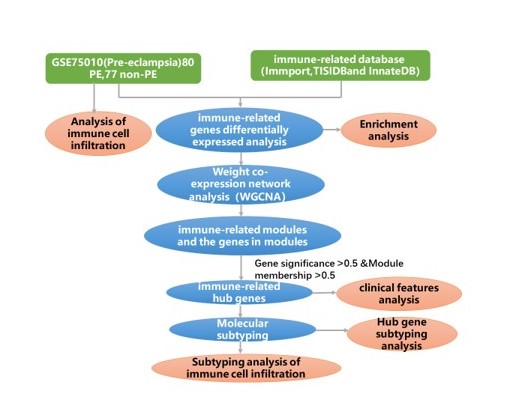

Supplement: Supplemental Material [file KBIE_A_1875707_SM5546.zip › supplement/GraphicalAbstract.jpg]

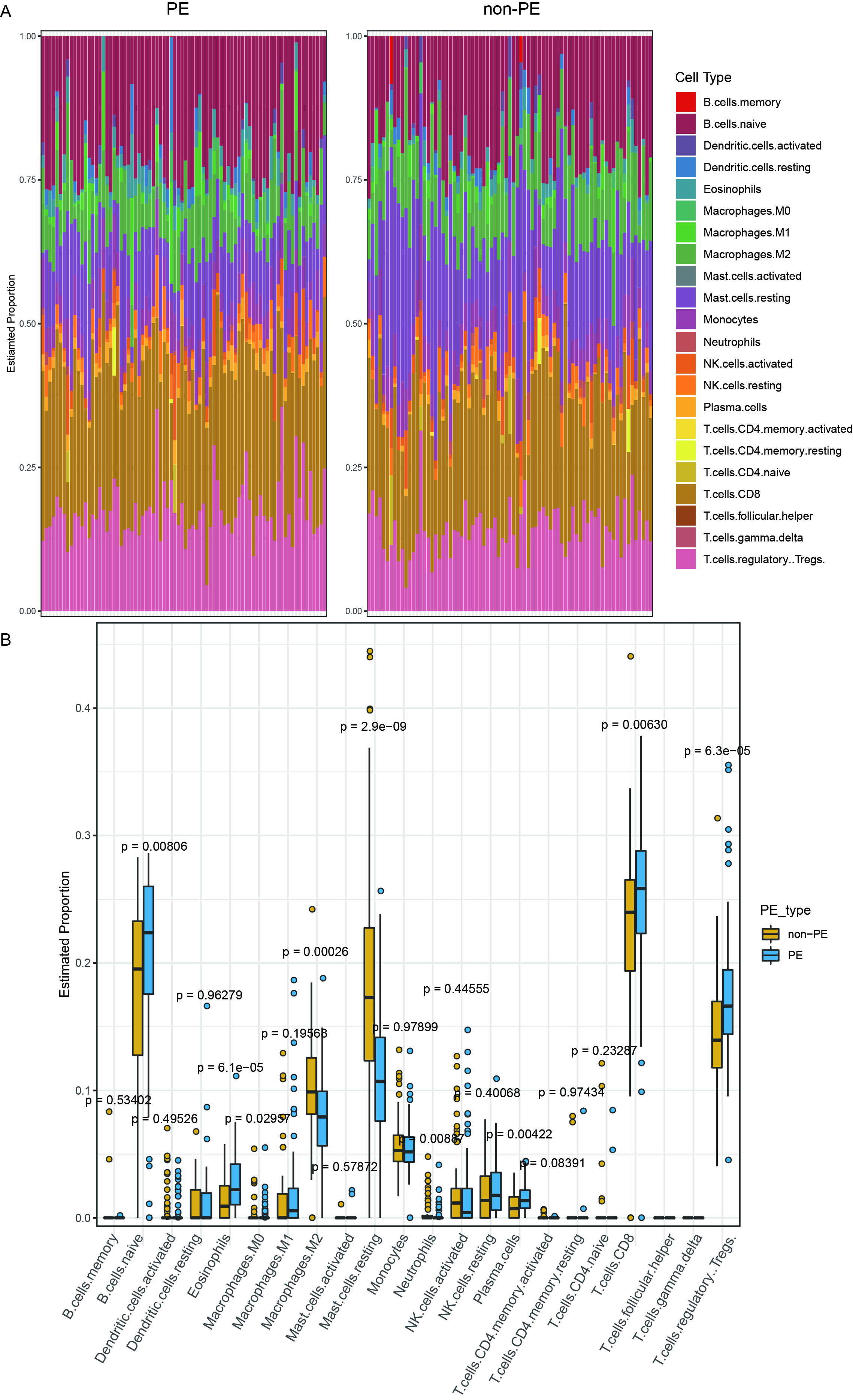

Supplement: Supplemental Material [file KBIE_A_1875707_SM5546.zip › supplement/Supplement Figure 1 300ppi.tif]
